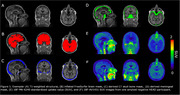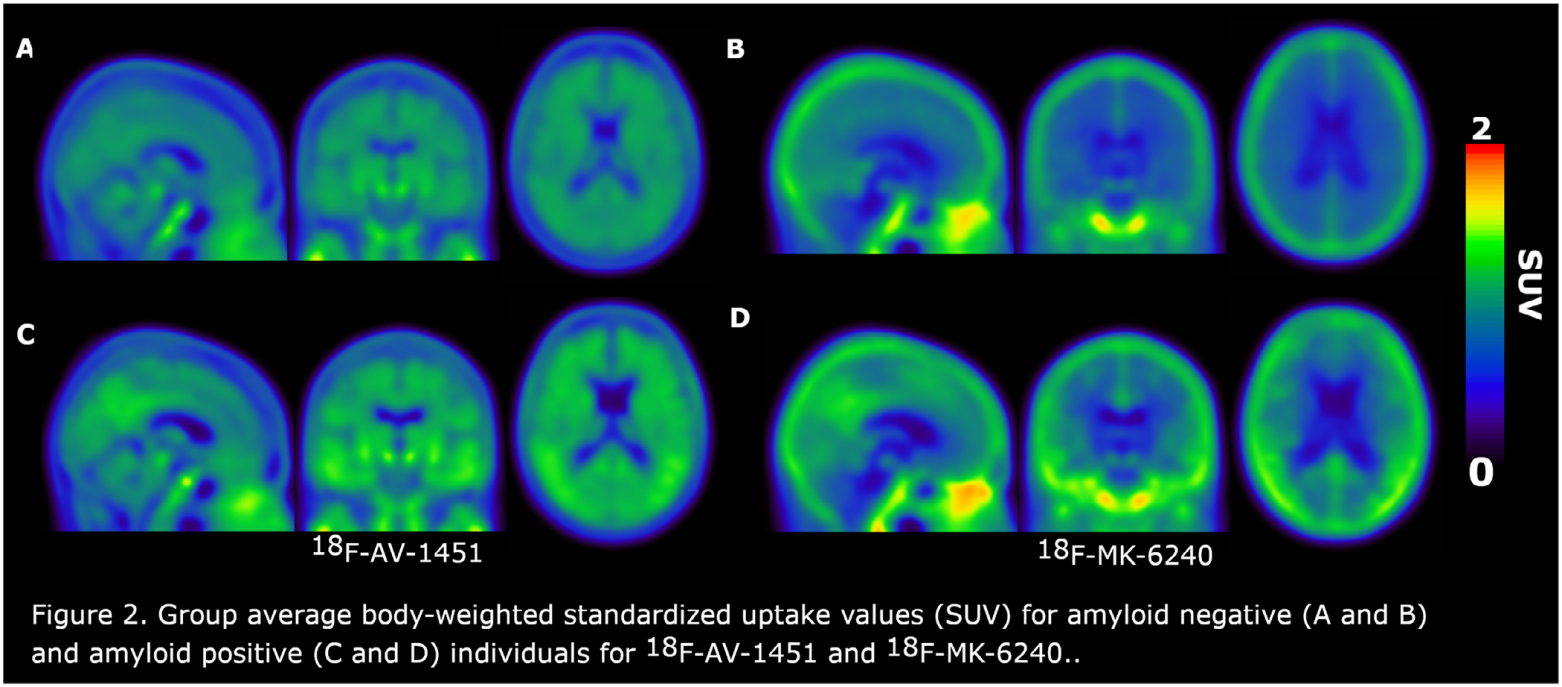# Comparing Off‐Target Meningeal and Skull Signal in Head‐to‐Head MK‐6240 and AV‐1451 Tau PET

**DOI:** 10.1002/alz70856_101657

**Published:** 2025-12-25

**Authors:** Shaney Flores, Thomas Hunter Smith, Jalen Scott, Danielle Gray, Yi Su, Diana A Hobbs, Sarah J. Keefe, Jacqueline Rizzo, Hope Shimony, Tammie L.S. Benzinger, David N. Soleimani‐Meigooni, Hwamee Oh, Juan Fortea, Belen Pascual, Pedro Rosa‐Neto, Tharick A Pascoal, Suzanne L. Baker, Brian A. Gordon, the HEAD Study

**Affiliations:** ^1^ Washington University School of Medicine, St. Louis, MO, USA; ^2^ Banner Alzheimer's Institute, Phoenix, AZ, USA; ^3^ Washington University School of Medicine in St. Louis, St. Louis, MO, USA; ^4^ Memory and Aging Center, Weill Institute for Neurosciences, University of California San Francisco, San Francisco, CA, USA; ^5^ Brown University, Providence, RI, USA; ^6^ Sant Pau Memory Unit, Department of Neurology, Hospital de la Santa Creu i Sant Pau, Institut d'Investigació Biomèdica Sant Pau (IIB SANT PAU), Facultad de Medicina ‐ Universitat Autònoma de Barcelona, Barcelona, Spain; ^7^ Houston Methodist Research Institute, Houston, TX, USA; ^8^ Douglas Mental Health University Institute, Montreal, QC, Canada; ^9^ Translational Neuroimaging Laboratory, The McGill University Research Centre for Studies in Aging, Montréal, QC, Canada; ^10^ University of Pittsburgh School of Medicine, Pittsburgh, PA, USA; ^11^ Lawrence Berkeley National Laboratory, Berkeley, CA, USA

## Abstract

**Background:**

Tau tangle deposition is associated with Alzheimer disease (AD) clinical symptomology and cognitive decline. Such deposition can be measured *in vivo* using positron emission tomography (PET). Off‐target signal from extra‐cerebral sources, such as skull or meninges, may bias quantification but the impact is currently unknown. Here, we investigate off‐target sources in individuals with crossover 18F‐AV‐1451 and 18F‐MK‐6240 tau PET scans within the HEAD study.

**Method:**

T1‐weighted magnetic resonance imaging (MRI), Pittsburgh Compound B (PiB) amyloid PET, and tau PET were acquired for 42 participants. Tau PET scans were quantified using body‐weighted standardized uptake values (SUV) and SUV ratios (SUVRs) with cerebellar grey as the reference region. To isolate skull bone, CT scans from each participant were aligned to their own T1 image and threshold using a Hounsfield units cutoff derived from applying Gaussian mixture modeling on the average of all CT images to identify a skull bone compartment. For meninges, MNI‐152 aligned 18F‐MK‐6240 SUVR images were averaged and thresholded to identify voxels that potentially were meninges. This template was then transformed back into participant space and masked using the skull bone image and a brain mask to isolate those voxels that were neither bone nor brain but fell in‐between, producing a subject‐specific meningeal mask (Figure 1). Group average SUV images were created between amyloid negative and positive individuals.

**Result:**

Average age of participants was 66 (21‐88) years. 25 were biologically female and 15 were amyloid positive. Group average SUV images show extra‐cerebral off‐target signal was more pronounced in 18F‐MK‐6240 for both amyloid negative and positive individuals compared to 18F‐AV‐1451 (Figure 2). The extra‐cerebral 18F‐MK‐6240 signal appeared across the entire cortical surface and posterior cerebellum, potentially impacting a cerebellar reference for tau PET SUVR quantification.

**Conclusion:**

While off‐target extra‐cerebral signal appears in both tau PET tracers, it was more pronounced and wide‐spread in 18F‐MK‐6240. Subject‐specific skull and meningeal masks can parse this signal to aid in cause determination. Additional work is needed to explore factors contributing to this signal, such as demographic, health, and genetic.